# Multivariate Analyses of Balance Test Performance, Vestibular Thresholds, and Age

**DOI:** 10.3389/fneur.2017.00578

**Published:** 2017-11-08

**Authors:** Faisal Karmali, María Carolina Bermúdez Rey, Torin K. Clark, Wei Wang, Daniel M. Merfeld

**Affiliations:** ^1^Jenks Vestibular Physiology Laboratory, Mass Eye and Ear Infirmary, Boston, MA, United States; ^2^Otolaryngology, Harvard Medical School, Harvard University, Boston, MA, United States; ^3^Smead Aerospace Engineering Sciences, University of Colorado, Boulder, CO, United States; ^4^Division of Sleep Medicine, Brigham and Women’s Hospital, Boston, MA, United States

**Keywords:** vestibular, balance, perception, thresholds, aging, multivariate

## Abstract

We previously published vestibular perceptual thresholds and performance in the Modified Romberg Test of Standing Balance in 105 healthy humans ranging from ages 18 to 80 (1). Self-motion thresholds in the dark included roll tilt about an earth-horizontal axis at 0.2 and 1 Hz, yaw rotation about an earth-vertical axis at 1 Hz, y-translation (interaural/lateral) at 1 Hz, and z-translation (vertical) at 1 Hz. In this study, we focus on multiple variable analyses not reported in the earlier study. Specifically, we investigate correlations (1) among the five thresholds measured and (2) between thresholds, age, and the chance of failing condition 4 of the balance test, which increases vestibular reliance by having subjects stand on foam with eyes closed. We found moderate correlations (0.30–0.51) between vestibular thresholds for different motions, both before and after using our published aging regression to remove age effects. We found that lower or higher thresholds across all threshold measures are an individual trait that account for about 60% of the variation in the population. This can be further distributed into two components with about 20% of the variation explained by aging and 40% of variation explained by a single principal component that includes similar contributions from all threshold measures. When only roll tilt 0.2 Hz thresholds and age were analyzed together, we found that the chance of failing condition 4 depends significantly on both (*p* = 0.006 and *p* = 0.013, respectively). An analysis incorporating more variables found that the chance of failing condition 4 depended significantly only on roll tilt 0.2 Hz thresholds (*p* = 0.046) and not age (*p* = 0.10), sex nor any of the other four threshold measures, suggesting that some of the age effect might be captured by the fact that vestibular thresholds increase with age. For example, at 60 years of age, the chance of failing is roughly 5% for the lowest roll tilt thresholds in our population, but this increases to 80% for the highest roll tilt thresholds. These findings demonstrate the importance of roll tilt vestibular cues for balance, even in individuals reporting no vestibular symptoms and with no evidence of vestibular dysfunction.

## Introduction

Deficits in postural control and resulting falls have profound public health implications (>31,000 deaths and >800,000 hospitalizations/year) (2). Sensory feedback plays a critical role in postural control (3–10). A number of studies have linked vestibular function and falls. One epidemiological study (11) showed that that failure to complete the condition 4 of the Modified Romberg foam test (12–14), which is commonly considered a balance assay of vestibular function, was highly correlated with “difficulty with falling” in the past 12 months. Another epidemiological study reported that 80% of fallers admitted to an Emergency department in the UK had symptoms of vestibular impairment (15). Most recently, we reported significant correlations between failure to complete the Modified Romberg foam balance test and roll tilt perceptual thresholds in the dark (1). These roll tilt perceptual thresholds were previously shown to primarily be a measure of vestibular function (16). These findings complement previous studies showing that vestibular dysfunction negatively impacts clinical balance test performance (3, 4, 6, 17–20). While speculative, a previous manuscript (1) also provided statistical calculations that emphasize the significance of the problem, suggesting that falls associated with vestibular function might cause somewhere between 50,000 and 150,000 deaths in America each year. This would rank vestibular dysfunction somewhere between number 10 and number 3 on the list of leading causes of death in the United States^1^ (21).

The aforementioned studies emphasize the importance of understanding the connections between vestibular function (and dysfunction), age, and falls. Therefore, we decided to perform another set of analyses on our previously published data set (1) of 105 subjects between 18 and 80 year of age who had been prescreened to be suffering no vestibular symptoms. There was no evidence for differences in the thresholds of males and females, but statistically significant threshold increases above the age of 40 were reported for all five motions investigated: roll tilt about an earth-horizontal axis at 0.2 and 1 Hz, yaw rotation about an earth-vertical axis at 1 Hz, y-translation (interaural/lateral) at 1 Hz, and z-translation (vertical) at 1 Hz. These threshold data were best modeled by a two-segment age model having a constant baseline below an age cutoff around 40 years of age with thresholds increasing above the age of 40. Building on these findings, this manuscript focuses on correlations—both correlations in the threshold measures themselves and correlations between thresholds, age, and failing the vestibular part (condition 4) of the Modified Romberg foam test.

Correlations between thresholds and balance are important because they might help us intervene to prevent falls *via* (a) warnings (e.g., verbal) from a clinician informing patients about their individual fall risk, (b) rehabilitation/training (22, 23), or (c) *via* the use of prosthetics/aids like canes, balance feedback devices (24), vestibular prosthesis (25–28), vibro-tactile shoes (29), stochastic resonance of the vestibular system (30–33), and/or orthotic devices (34).

Understanding the correlations between thresholds for different types of motion (e.g., yaw rotation and interaural translation) is also important as it could provide clues regarding the mechanism that causes increased thresholds with age (1). To further explore this, we used principal component analysis (PCA) to understand the structure underlying intersubject variability in thresholds. Moreover, given the vital importance that thresholds might play in predicting fall risk, understanding correlations between thresholds may improve fall risk evaluation.

As detailed in Section “Discussion,” these multivariate analyses yield new findings that were not presented in our previous study. First, while our previous univariate analysis found that all five threshold measures were correlated with chance of failing condition 4, these new multivariate analyses show that only roll tilt 0.2 Hz has a statistically significant correlation after taking into account the relationship between the threshold measures. While the previous analyses found that thresholds are correlated with chance of failing condition 4 even after adjusting for age effects, the new analyses show how the chance of failing depends on the combination of age and roll tilt 0.2 Hz thresholds. We provide correlation coefficients between all threshold measures both before and after age adjustment. Furthermore, new analyses determine the structure of variation in thresholds, including the effects of aging and individual differences.

## Materials and Methods

### Subjects

As previously described (1), 105 subjects (54 females, 51 males) participated, ranging from 18 to 80 years old. Subjects were excluded if they reported, *via* a questionnaire, any major health problems (e.g., a history of neurological, otologic, vestibular, and chronic uncontrolled diseases) or any history of vestibular symptoms. The study was approved by the MEEI Human Studies Committee, and written informed consent was obtained from all subjects as dictated by the Declaration of Helsinki.

### Balance Testing

The modified Romberg test of standing balance on firm and compliant support surfaces (14) was used to assess balance function (12, 13). Subjects stand with arms crossed and feet together. The test has four conditions, each of which was scored as pass/fail. Each condition must be passed to progress to the next, with two attempts permitted for each condition, according to the following criteria. In condition 1, the subject must stand on the floor for 15 s with eyes open. In condition 2, the subject must stand on the floor for 15 s with eyes closed. In condition 3, the subject must stand on memory foam with eyes open for 30 s. In condition 4, the subject must stand on the foam with eyes closed for 30 s. A fail occurred when subjects did any of the following before the allotted time for each trial: move their feet for stability, open their eyes, or release their arms. Condition 4 primarily assesses vestibular function (11, 14), since visual contributions are eliminated and the foam makes kinesthetic cues unreliable. Six subjects did not perform the balance test and were not included in the balance analyses in this study.

### Vestibular Threshold Measurements

The detailed methods used to assay perceptual threshold have been published (1, 16, 35, 36). Upright subjects in the dark were seated in a chair with a helmet to reduce head movement and a five-point harness. The chair was mounted on a Moog 6DOF motion platform that delivered single-cycle acceleration motion stimuli. Subjects listened to white noise through noise-canceling headphones, both to indicate when motion was occurring and to mask other auditory cues. To improve efficiency (37), stimuli magnitudes were selected using an adaptive three-down, one-up staircase (37, 38), in which stimuli become smaller after three consecutive correct responses, and larger after one incorrect response. The staircase followed parameter estimation by sequential testing (PEST) rules (39). The direction (e.g., left/right) was determined randomly for each trial. Testing occurred in blocks of 100 trials, and each trial consisted of a motion stimulus followed by a response. Subjects reported their perception of motion direction using buttons in their left or right hand and were required to report a perception for every trial.

Each subject participated in five blocks of testing lasting approximately 3 h—one block for each of the five types of motion—with the following “motion type” conditions: (1) “yaw 1 Hz”—yaw rotations about an earth-vertical axis at 1 Hz (i.e., a motion duration of 1 s), with an initial stimulus of 4°/s; (2) “Y 1 Hz”—y-translations (lateral/interaural) along an earth-horizontal axis at 1 Hz, with an initial stimulus of 4 cm/s; (3) “Z 1 Hz”—z-translations (superior–inferior) along an earth-vertical axis at 1 Hz, with an initial stimulus of 16 cm/s; (4) “Roll tilt 1 Hz”—ear-down tilts about a head-centered earth-horizontal axis at 1 Hz, with an initial stimulus of 3°/s; and (5) “Roll tilt 0.2 Hz”—the same at 0.2 Hz, with an initial stimulus of 2°/s. Motions at 1 Hz were selected to focus on either the semicircular canals (SCCs) or otolith organs and because 100 trials can be completed in less than 10 min; roll tilt 0.2 Hz was used to study integration of otolith and SCC cues (40).

Threshold (σ) was determined for each block by fitting a Gaussian cumulative distribution psychometric function (41, 42) to the binary responses. The mean of the Gaussian (μ) is often called the “vestibular bias” and corresponds to the stimulus for which there is an equal likelihood of a left vs. right response (or up vs. down).

### Data Analysis

All analyses were performed using Matlab R2014b (Mathworks, MA, USA). All statistical analyses were conducted using log-transformed thresholds (e.g., geometric means for across subject averages) because vestibular thresholds have been shown to demonstrate a lognormal distribution across subjects (1, 43, 44).

Correlation coefficients were calculated using Pearson’s correlation. When statistical tests were performed on correlations across the thresholds for different motion types, yielding ten statistical tests, a Bonferroni correction was used for multiple comparisons, using a critical value (*p*_c_) of 0.05/10 = 0.005. PCA was performed after standardizing log-transformed thresholds by subtracting the mean from all values and dividing by the SD.

Multiple variable logistic regression was used to model the relationship between the chance of failing condition 4 of the balance test and thresholds, age and sex; logistic regression was used because of the binary pass/fail nature of the dependent variable. The Matlab command fitglm with a binomial distribution was used to perform logistic regressions. Stepwise regression (45) was used for variable selection using the Matlab command stepwiseglm, which uses a forward and backward stepwise procedure and a Bayesian information criteria (BICs). All regressions included an intercept term, which was statistically significant in all analyses.

Analyses were also performed on age-adjusted thresholds. Our previous publication (1) determined that a piecewise model best described the effect of aging on vestibular thresholds, with a constant baseline below 42.5 years, followed by a linear increase above this age cutoff. Each motion type had unique baseline and slope values. For subjects younger than the age cutoff, the age-adjusted threshold was simply their threshold. For subjects older than the age cutoff, we determine each age-adjusted threshold using this equation:

age adjusted threshold=elog(​threshold) − log(​slope*[​age − cutoff] + baseline​) + log(​baseline​)=threshold⋅baselineslope⋅[age−cutoff]+baseline.

Effectively, this factored out known changes with age by modifying any threshold above the age cutoff—leaving only threshold variations after removing the effect of age. Note that subtraction of the threshold from the model is done after log transformation because of the lognormal threshold distribution.

## Results

### Threshold Correlations

#### Thresholds

**Figure 1:**
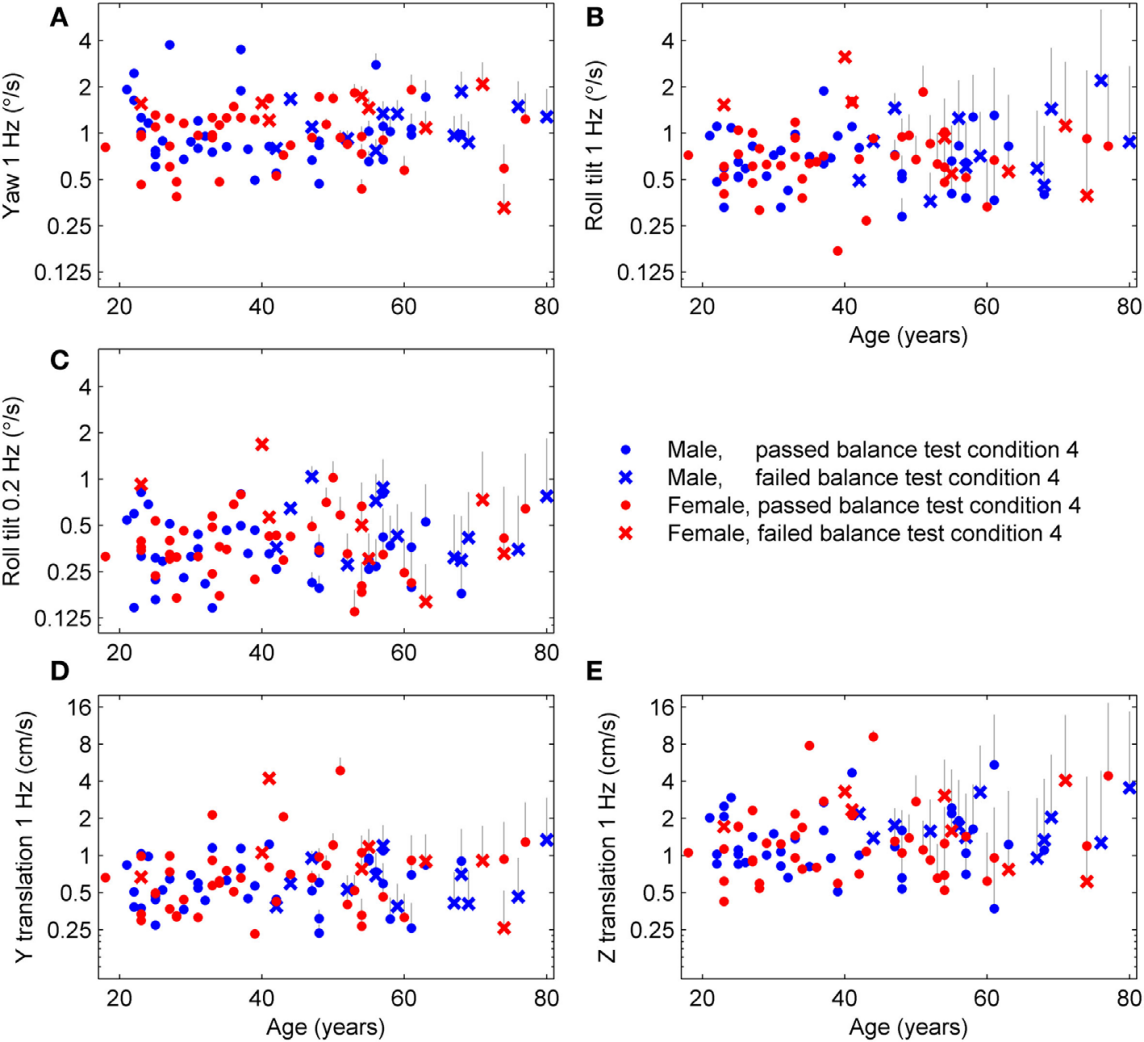
Age-adjusted thresholds for each subject and each of the five motion types. The published aging regression (1) found that thresholds had a constant baseline until 42.5 years, then increased at different rates for each of the five threshold measures. Thresholds for each subject above 42.5 years were adjusted to leave only variations independent of age. **(A)** The gray lines show the decrease in yaw threshold applied to each subject, with the top of the line showing the uncorrected threshold and the symbol at the bottom of the line showing the age-adjusted threshold. **(B–E)** Results for the four other threshold measures.

We applied the published age fitting model (1) to compute the age-adjusted thresholds (Figure 1) which are simply the thresholds after removing the average age effect. Results are segmented by sex to illustrate that there is no effect of sex even after age adjustment, which confirms our previous report of no statistically significant differences between females and males in any threshold measure. Results are also segmented by subjects who passed (⚪) and failed (×) condition 4 of the balance test and show that subjects who failed condition 4 tended to have higher roll tilt thresholds. Older subjects also tended to fail condition 4 more than younger subjects (i.e., more failures on right side of plots).

**Figure 2:**
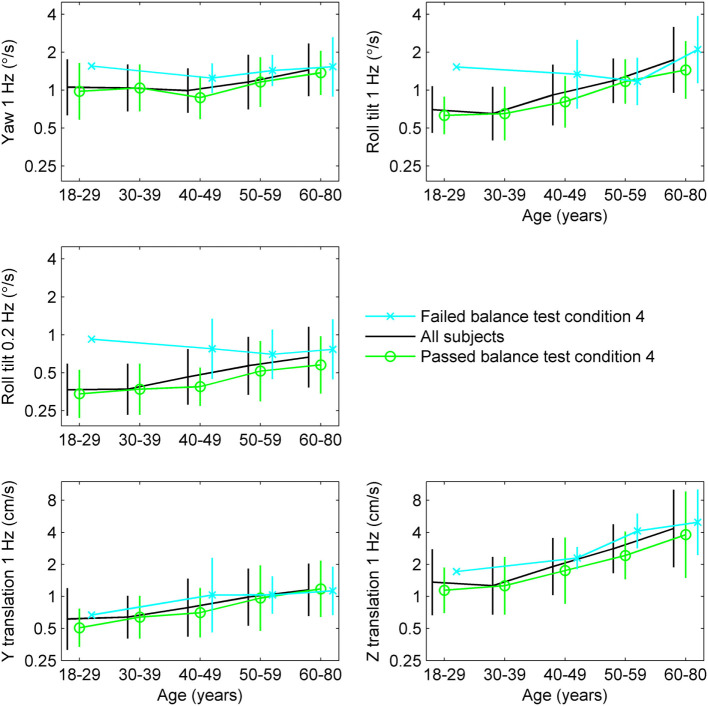
Thresholds segmented by age group and results of condition 4 of the balance test, with error bars showing SD.

To further explore the relationship between age, thresholds and balance, we determined the average thresholds for different age groups segmented by results of condition 4 of the balance test (Table 1; Figure 2). While detailed statistical analyses of this relationship will follow, we note a tendency toward higher thresholds for subjects who failed condition 4 in comparison to those who passed condition 4.

**Table 1:** Thresholds segmented by age group and results of condition 4 of the balance test, with 95% confidence intervals.

Age (years)	No. subjects	Yaw 1 Hz (°/s)	y-translation 1 Hz (cm/s)	z-translation 1 Hz (cm/s)	Roll tilt 1 Hz (°/s)	Roll tilt 0.2 Hz (°/s)
All	105	1.11 (1.01–1.23)	0.78 (0.69–0.89)	1.97 (1.68–2.30)	0.93 (0.83–1.04)	0.46 (0.41–0.51)
18–29	29	1.06 (0.87–1.28)	0.61 (0.48–0.79)	1.36 (1.04–1.77)	0.70 (0.60–0.82)	0.37 (0.31–0.44)
30–39	20	1.04 (0.86–1.26)	0.64 (0.52–0.79)	1.26 (0.95–1.67)	0.65 (0.52–0.81)	0.37 (0.30–0.46)
40–49	19	0.99 (0.83–1.20)	0.79 (0.59–1.05)	1.91 (1.44–2.53)	0.92 (0.71–1.18)	0.46 (0.37–0.59)
50–59	21	1.16 (0.93–1.44)	0.99 (0.75–1.29)	2.81 (2.23–3.53)	1.19 (1.00–1.42)	0.57 (0.45–0.72)
60–80	16	1.45 (1.14–1.85)	1.15 (0.87–1.53)	4.35 (2.86–6.61)	1.74 (1.29–2.35)	0.67 (0.50–0.88)

Passed balance	79	1.04 (0.94–1.16)	0.69 (0.61–0.79)	1.62 (1.37–1.92)	0.81 (0.71–0.91)	0.40 (0.36–0.45)
18–29	24	0.98 (0.79–1.21)	0.51 (0.43–0.60)	1.14 (0.93–1.40)	0.63 (0.55–0.73)	0.34 (0.29–0.41)
30–39	20	1.04 (0.86–1.26)	0.64 (0.52–0.79)	1.26 (0.95–1.67)	0.65 (0.52–0.81)	0.37 (0.30–0.46)
40–49	13	0.87 (0.70–1.09)	0.70 (0.52–0.95)	1.75 (1.18–2.60)	0.81 (0.62–1.05)	0.39 (0.32–0.47)
50–59	14	1.16 (0.91–1.48)	0.97 (0.66–1.41)	2.43 (1.84–3.19)	1.17 (0.94–1.46)	0.52 (0.38–0.69)
60–80	8	1.37 (1.03–1.83)	1.18 (0.77–1.81)	3.80 (1.97–7.35)	1.45 (1.00–2.10)	0.58 (0.40–0.84)

Failed balance	20	1.43 (1.19–1.71)	1.05 (0.81–1.35)	3.67 (2.79–4.84)	1.55 (1.18–2.04)	0.76 (0.60–0.95)
18–29	1	1.56 (1.56–1.56)	0.67 (0.67–0.67)	1.71 (1.71–1.71)	1.53 (1.53–1.53)	0.92 (0.92–0.92)
30–39	0	–	–	–	–	–
40–49	5	1.25 (0.98–1.58)	1.03 (0.50–2.11)	2.29 (1.85–2.84)	1.34 (0.76–2.34)	0.78 (0.48–1.27)
50–59	6	1.43 (1.14–1.81)	1.03 (0.74–1.44)	4.12 (3.04–5.59)	1.18 (0.83–1.67)	0.70 (0.49–1.01)
60–80	8	1.53 (1.05–2.25)	1.13 (0.78–1.63)	4.97 (3.02–8.19)	2.10 (1.36–3.23)	0.77 (0.52–1.13)

#### Correlations between Vestibular Threshold Measures

**Figure 3:**
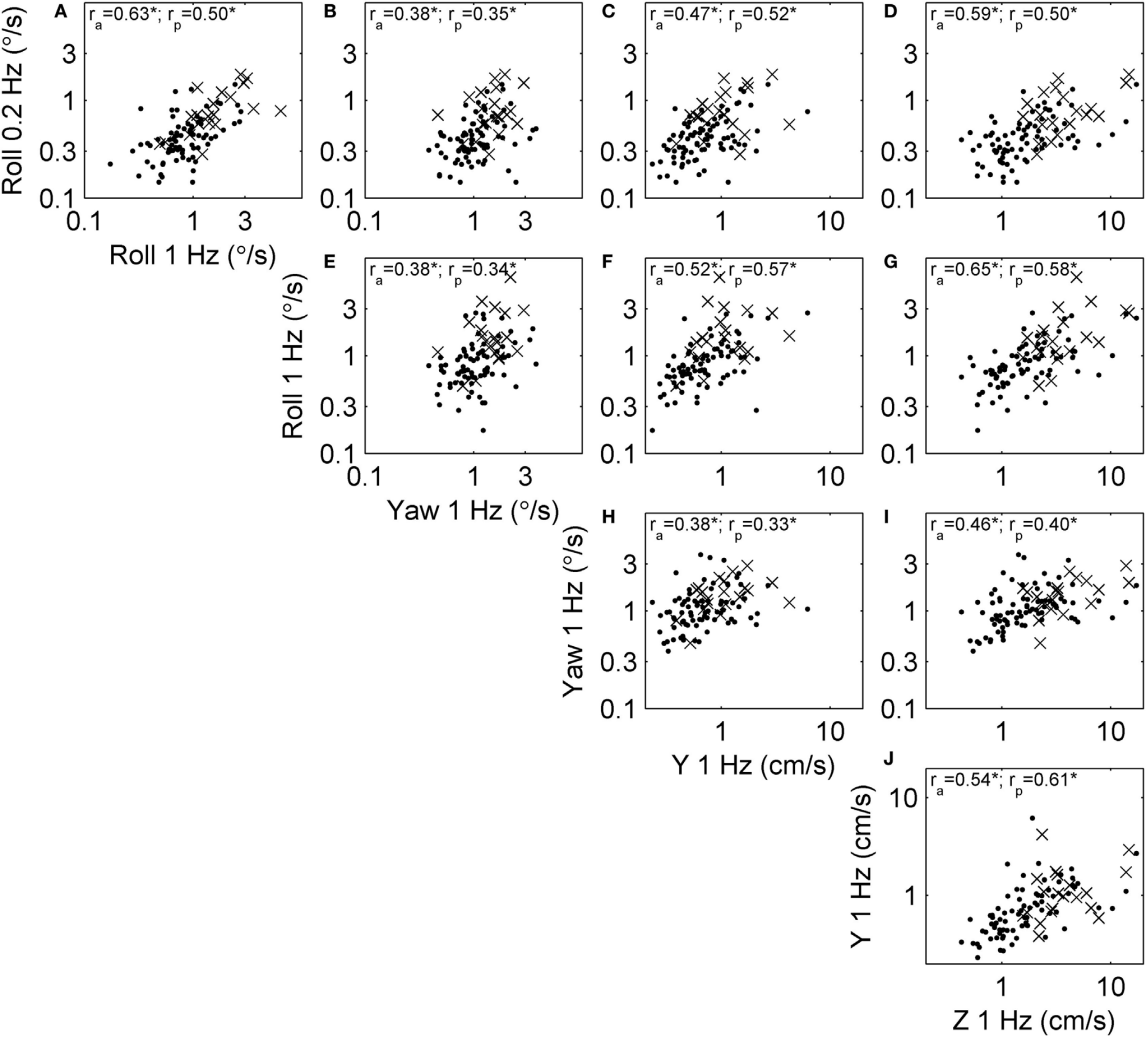
Scatterplots showing the relationship between each of the threshold measures across subjects. Each subject is shown, and segmented into those who passed (dots) and failed (×) condition 4 of the balance test. Correlation coefficients were calculated using log-transformed thresholds, including the correlation across all subjects (*r*_a_) and only those who passed condition 4 (*r*_p_). All correlations were significant at the *p* < 0.005 level (indicated by *). **(A)** The relationship between roll tilt 0.2 Hz thresholds and roll tilt 1 Hz thresholds across subjects. **(B–J)** The relationships between other corresponding pairs of thresholds.

We examined correlations between threshold measures for each motion type, since these correlations have important practical implications and also provide some insight into the shared organs and mechanisms underlying vestibular sensation. Figure 3 shows the relationship between thresholds across subjects. For example, Figure 3A shows the relationship between roll tilt 0.2 Hz thresholds and roll tilt 1 Hz thresholds. In this case, the correlation coefficient was *r*_a_ = 0.63 for all subjects, and *r*_p_ = 0.50 for only subjects who passed condition 4 of the balance test. Each threshold pair had a positive, statistically significant (multiple comparison, *p* < 0.005) correlation.

**Figure 4:**
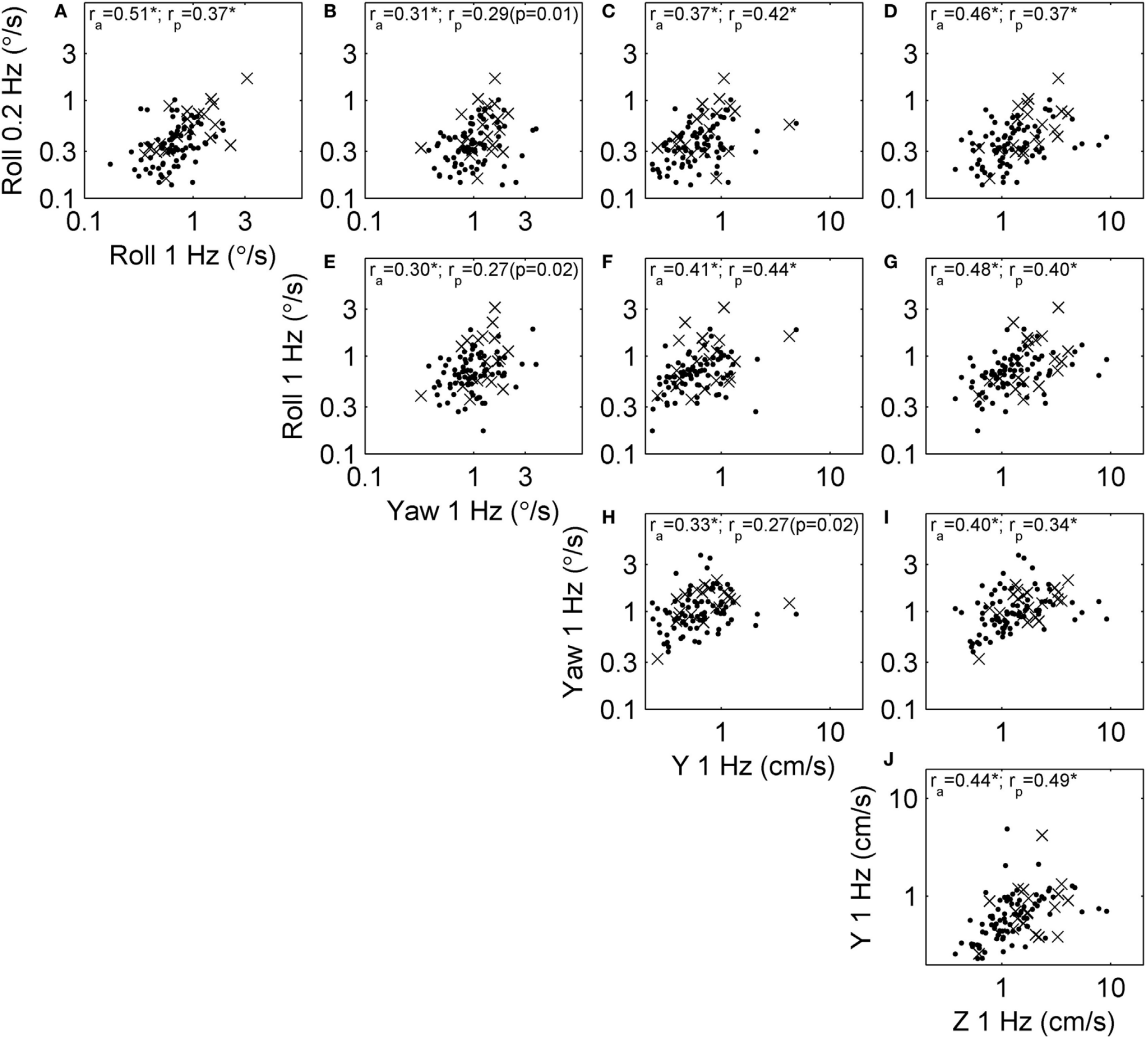
Scatterplots showing the relationship between each of the age-adjusted threshold measures across subjects. This repeats that analysis shown in Figure 3 in a way that focuses on correlation due to factors other than aging. Each subject is shown, and segmented into those who passed (dots) and failed (×) condition 4 of the balance test. Correlation coefficients were calculated using log-transformed, age-adjusted thresholds, including the correlation across all subjects (*r*_a_) and only those who passed condition 4 (*r*_p_). Most correlations were statistically significant at the *p* < 0.005 level (indicated by *), with exceptions noted by the actual *p* value. **(A)** The relationship between roll tilt 0.2 Hz thresholds and roll tilt 1 Hz thresholds across subjects. **(B–J)** The relationships between other corresponding pairs of thresholds.

Since all thresholds increase with age, some correlations may occur because of the aging process. To focus on correlation due to factors other than aging, we performed the same analysis on age-adjusted thresholds (Figure 1). Figure 4 shows the relationships between these age-adjusted thresholds. All correlation coefficients decreased somewhat (Table 2) compared with unadjusted thresholds, but all remained statistically significant (multiple comparison, *p* < 0.005) when calculated for all subjects. Thus, age alone does not explain these correlations. Section “Discussion” provides our interpretation of these correlation coefficients, including the observation that the lowest correlation coefficients were between yaw thresholds and the other four thresholds. When calculated for subjects who passed condition 4, most correlation coefficients were statistically significant, with a few exceptions (*p* = 0.01, *p* = 0.02, *p* = 0.02) that did not reach statistical significance after multiple comparisons correction (*p*_c_ of 0.05/10 = 0.005).

**Table 2:** The reduction in correlation coefficients due to age adjustment of thresholds.

	Roll 1 Hz	Yaw	Y	Z
Roll 0.2 Hz	−27% (0.63 to 0.51)	−17% (0.38 to 0.31)	−21% (0.47 to 0.37)	−27% (0.59 to 0.46)
Roll 1 Hz		−21% (0.38 to 0.30)	−24% (0.52 to 0.41)	−31% (0.65 to 0.48)
Yaw			−14% (0.38 to 0.33)	−12% (0.46 to 0.40)
Y				−18% (0.54 to 0.44)

*Differences shown for coefficients calculated across all subjects (*r*_a_)*.

#### Structure Underlying Variation in Vestibular Thresholds

We examined the underlying structure of the relationship between thresholds. This was done using PCA, which determines principal components that are a weighted combination of each of the other measures such that they explain most of the variation within the data. The PCA analysis yielded similar results for unadjusted thresholds (Table 3) and age-adjusted threshold (Table 4). The contribution shows the variation in the data explained by each component. Since age adjustment explains 20.7% of the variation in the data (1), we also determined the variation explained by each component as a fraction of the remaining 79.3% of the variation (Table 5, second row). For comparison, the variation explained by each component for the unadjusted thresholds is shown (Table 5, first row).

**Table 3:** Principal component analysis (PCA) of log-transformed thresholds for all subjects.

	First	Second	Third	Fourth	Fifth
Contribution (%)	**60**	14	11	8	7
Yaw	**0.37**	0.90	−0.16	0.08	0.13
Y	**0.43**	−0.04	0.87	0.22	0.02
Z	**0.49**	−0.07	−0.07	−0.62	−0.60
Roll tilt 1 Hz	**0.48**	−0.31	−0.19	−0.29	0.75
Roll tilt 0.2 Hz	**0.46**	−0.29	−0.41	0.69	−0.25

*The five components are listed (first, second, etc.). The contribution shows the variation in the data explained by each component. The coefficients show the weight given to each threshold when combined to form each component. Thresholds were standardized before PCA. The component with the largest contribution is shown in bold*.

**Table 4:** Principal component analysis of age-adjusted, log-transformed thresholds for all subjects.

	First	Second	Third	Fourth	Fifth
Contribution (%)	**52**	15	13	10	10
Yaw	**0.38**	0.84	−0.29	−0.22	0.15
Y	**0.43**	0.03	0.85	−0.28	−0.07
Z	**0.48**	0.05	0.00	0.82	−0.30
Roll tilt 1 Hz	**0.47**	−0.40	−0.15	0.03	0.77
Roll tilt 0.2 Hz	**0.46**	−0.38	−0.41	−0.45	−0.53

*The component with the largest contribution is shown in bold*.

**Table 5:** Variance explained by each principal component after considering the variance explained by age adjustment.

	Age	First	Second	Third	Fourth	Fifth
Thresholds (%)	–	**60**	14	11	8	7
Age-adjusted thresholds (%)	21	**41**	12	10	8	8

*The first row shows the results for log-transformed thresholds without age adjustment. The second row shows the results for age-adjusted, log-transformed thresholds. The component with the largest contribution is shown in bold*.

More than half of intersubject variability is explained by the first component, suggesting a relatively low-dimensional structure underlying thresholds and vestibular sensory precision. Its projection remains (i.e., the weights remain) surprisingly similar after age adjustment, suggesting that lower or higher thresholds across all measures are an important individual trait. For unadjusted thresholds, the first component has a contribution of 60% (Table 3). After age adjustment, the first component has a contribution of 52% (Table 4), which is 41% (Table 5) of the total variance (52%⋅79.3% = 41%). This shows that much of the variation due to age was included in the first principal component. The remaining components are more difficult to interpret, but the second component may allow a decoupling of yaw and roll thresholds, concomitant with their low correlation.

### Correlations with Condition 4 of the Modified Romberg Foam Test

#### The Relationship between Vestibular Thresholds and Condition 4 of the Modified Romberg Foam Test

Our previous study (1) examined the basic relationship between failing condition 4 of the Modified Romberg test and vestibular thresholds. However, those analyses only looked at the relationship between each individual threshold and the chance of failing the test, without performing multivariate analyses. Specifically, we performed single-variable logistic regressions between failures and each age-adjusted threshold. There were statistically significant correlations with roll 0.2 Hz (*p* = 0.003) and roll 1 Hz (*p* = 0.02) thresholds, a suggestion of possible correlation with yaw 1 Hz (*p* = 0.09) and z-translation 1 Hz (*p* = 0.09) thresholds, and a non-significant correlation for y-translation (*p* = 0.50) thresholds. Given that thresholds may be correlated with each other, a weakness of this analysis was that it did not determine if the covariation between thresholds described above could have resulted in an artifact of some thresholds being correlated with the chance of failing condition 4.

**Figure 5:**
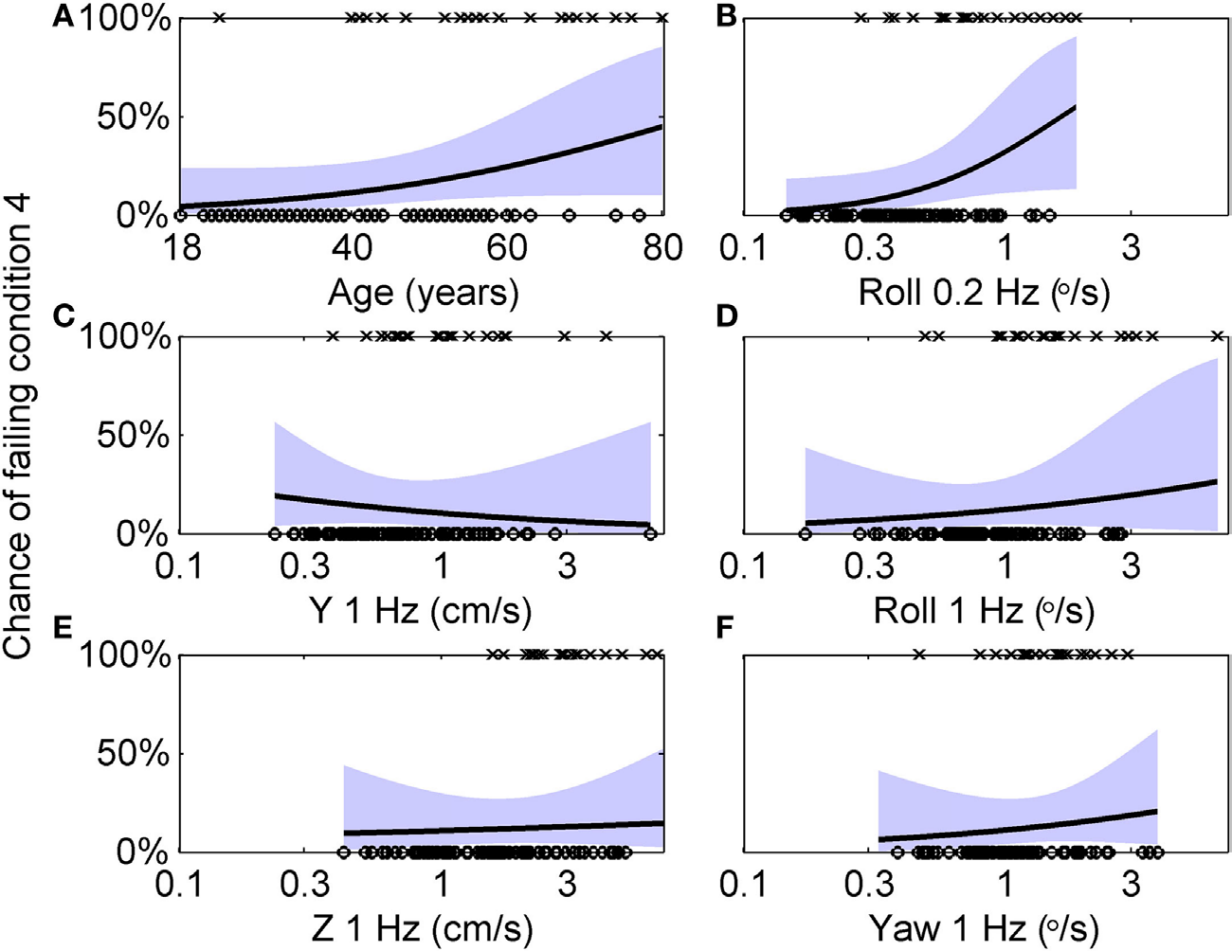
Logistic regression curves showing the dependency of failing condition 4 on age and each threshold measure. **(A)** Circles (○) indicate individual subjects who passed condition 4, while crosses (×) indicate subjects who failed condition 4. A logistic curve showing the dependency of the chance of failing condition 4 on age, with the shaded area showing the 95% confidence intervals. Each curve is generated by holding the other five variables at the median value of their sample, so it does not show the combined influence of multiple variables. **(B–F)** Similar analyses for each of the five threshold measurements.

Here, we further explore this relationship using multivariate analyses. The 99 subjects who did balance testing all passed conditions 1, 2, and 3. 79 subjects passed condition 4, while 20 failed. Table 6 shows the results of a multiple variable logistic regression to predict the chance of failing condition 4 based on age and vestibular thresholds. This analysis found that only roll tilt 0.2 Hz thresholds had a statistically significant (*p* = 0.046) relationship to the chance of failing condition 4. Sex and all other thresholds were not significant, with *p* > 0.47. In particular, roll tilt 1 Hz thresholds were not significantly associated with the chance of failing condition 4, even though our previous single-variable regression found a statistically significant relationship. This was likely due to the correlations between roll tilt 0.2 Hz and roll tilt 1 Hz thresholds described above, which demonstrates the importance of the multiple variable analysis. Figure 5 shows logistic regression curves depicting the dependency of failing condition 4 on age and each threshold measure. Figure 5A shows that younger subjects tend to pass (⚪) the balance test while older subjects tend to fail (×), with the logistic regression curve showing that the chance of failing the balance test is 4% for an 18 years old, and increases to 45% for an 80 years old. Each curve is generated by holding the other five variables at the median value of their sample, so it does not show the combined influence of multiple variables. Figure 5B shows that subjects with low roll tilt 0.2 Hz thresholds tend to pass (⚪) the balance test while subjects with higher thresholds tend to fail (×), with the logistic regression curve showing that the chance of failing the balance test is near 0% for the lowest roll tilt 0.2 Hz thresholds in our population, and increases to 61% for the highest thresholds. Figures 5C–F show the results for other thresholds, confirming the weak relationship between these variables and the chance of failing condition 4.

**Table 6:** Results of a multiple logistic regression to predict the chance of failing condition 4 of the balance test based on age and log-transformed vestibular thresholds.

	Estimate	SE	*t*-Stat	*p*-Value
(Intercept)	−3.24	1.39	−2.34	0.0193
Age	0.0457	0.0278	1.65	0.100
Sex	0.349	0.639	0.546	0.585
Yaw 1 Hz	0.543	0.763	0.711	0.477
Y 1 Hz	−0.484	0.700	−0.692	0.489
Z 1 Hz	0.173	0.600	0.289	0.773
Roll tilt 1 Hz	0.502	0.751	0.668	0.504
Roll tilt 0.2 Hz	1.51	0.757	2.00	0.0457*

*Signifies statistical significance (*p* < 0.05).

Since the regression revealed that many variables had little or no effect on the chance of failing condition 4, we aimed to create a simplified model that included only relevant variables. We used a stepwise procedure (45) to determine which variables to include in the model. Table 7 shows the results for the simplified model, which includes only age and roll tilt 0.2 Hz thresholds, both of which were statistically significant. This model had a lower BIC value than the complete model (85.4 vs. 106). To further confirm this model selection, we also investigated models that included: (1) each threshold without age, (2) age + each threshold, and (3) age + roll tilt 0.2 Hz thresholds + each other threshold. The selected model provided better fits than each of these, according to BIC values (these models are detailed in Section “Model Comparisons” in Appendix). We also considered a model that included age, roll tilt 0.2 Hz threshold and an interaction term between the two, but the interaction term was not significant (*p* = 0.054), and had slightly worse fit quality (BIC 85.8 vs. 85.4) so we focus on the simpler model.

**Table 7:** Results of a multiple logistic regression after application of a stepwise algorithm to provide a simplified model.

	Estimate	SE	*t*-Stat	*p*-Value
(Intercept)	−2.99	1.29	−2.32	0.0202
Age	0.0551	0.0222	2.48	0.0131*
Roll tilt 0.2 Hz	1.72	0.623	2.76	0.00584*

*Regression was performed on log-transformed thresholds*.

*Signifies statistical significance (*p* < 0.05).

**Figure 6:**
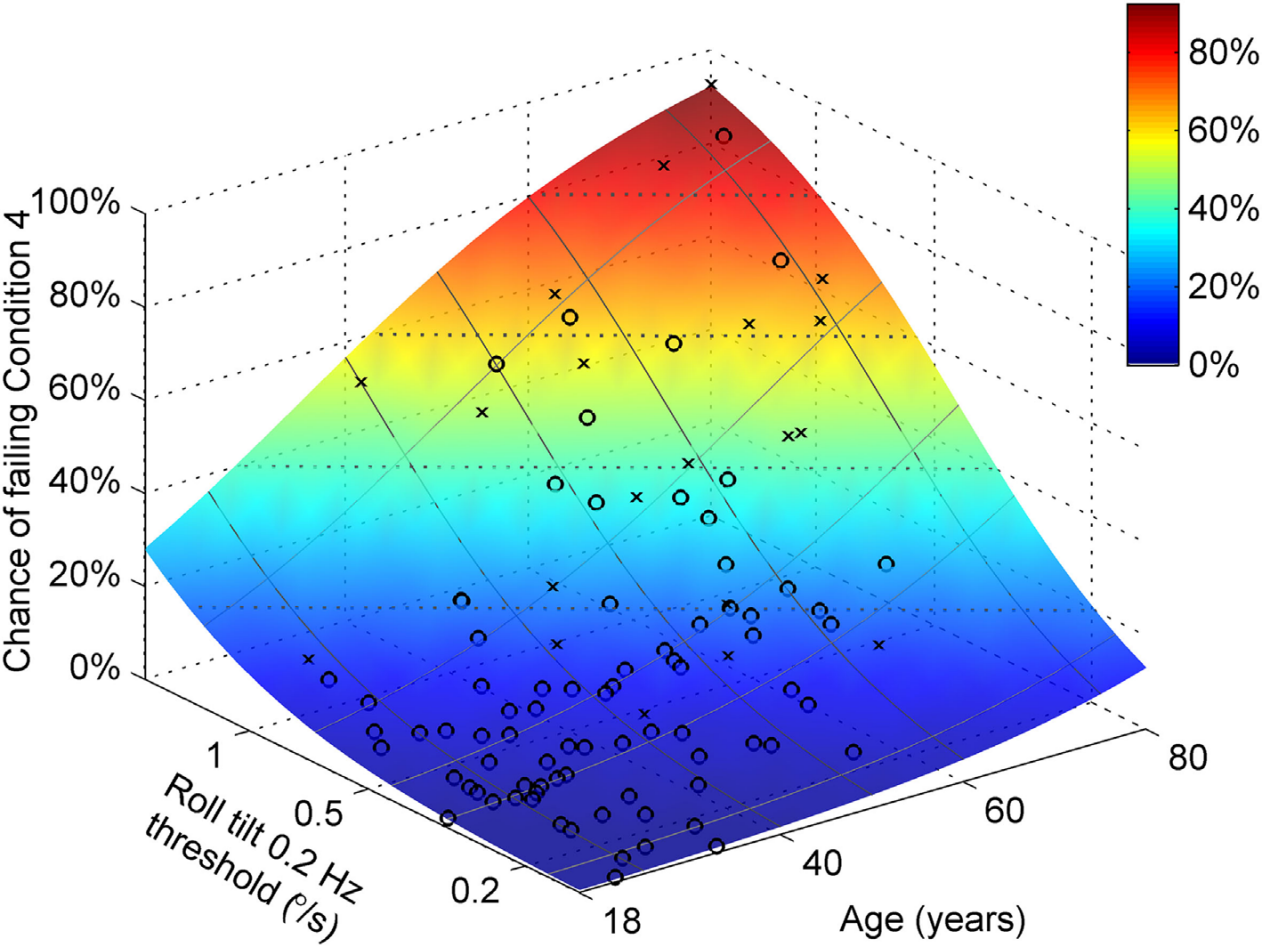
Logistic regression results showing the chance of failing condition 4 vs. both age and roll tilt 0.2 Hz thresholds determined using the model in Table 7. Symbols show subjects who passed (○) and failed (×) condition 4.

**Figure 7:**
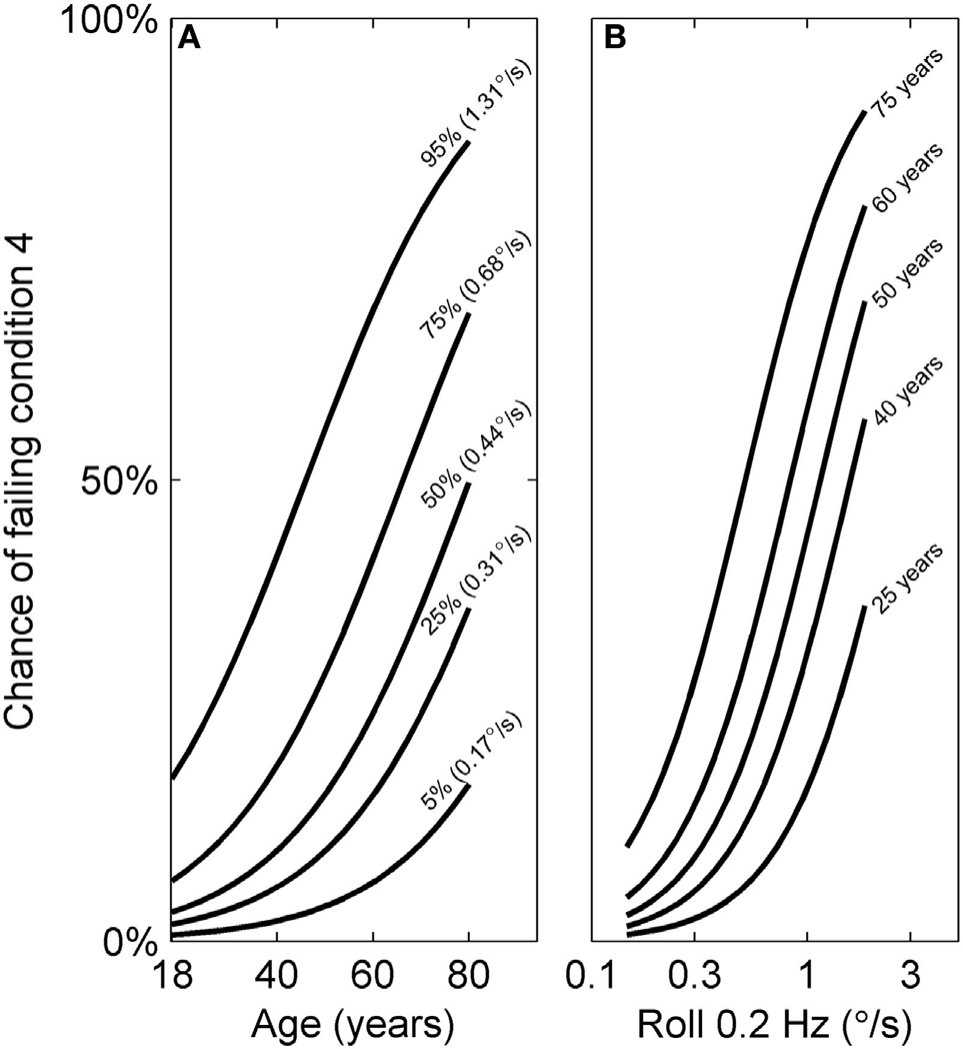
Logistic curves showing the chance of failing condition 4 vs. both age and roll tilt 0.2 Hz thresholds determined using the model in Table 7. **(A)** The dependency of failing condition 4 on age is shown for different roll tilt thresholds, which have been determined for the 5th, 25th, 50th, 75th, and 95th percentile within our sample. **(B)** The dependency of failing condition 4 on roll tilt 0.2 Hz thresholds for different ages (25, 40, 50, 60, and 75 years old). These curves are also shown in gray in Figure 6.

Both age and roll tilt 0.2 Hz thresholds had a statistically significant contribution to the chance of failing condition 4. Figure 6 shows the chance of failing condition 4 vs. both age and roll tilt 0.2 Hz thresholds. Generally it shows that the chance of failing condition 4 increases with both age and roll tilt 0.2 Hz thresholds. It emphasizes that for older subjects with high thresholds, the chance of failing is very high, approaching 90%. Figure 7A shows the dependency of failing condition 4 on age for different roll tilt thresholds. For example, for subjects whose roll tilt 0.2 Hz thresholds are in the 75th percentile, the chance of failing increases from 6% at age 18 to 68% at age 80. Figure 7B shows the dependency of failing condition 4 on roll tilt thresholds for subjects of different ages. For example, at 60 years old, the chance of failing is roughly 5% for the lowest roll tilt thresholds, which does not differ much from younger subjects. However, this increases to 80% for the highest roll tilt thresholds.

We also examined the relationship between the chance of failing condition 4 and age-adjusted thresholds shown in Figure 1. Table 8 shows the results of stepwise logistic regression using age-adjusted, log-transformed thresholds. Roll tilt 0.2 Hz thresholds and age were the only selected contributors and both were statistically significant, as for the unadjusted thresholds. While the coefficient for roll tilt 0.2 Hz thresholds was roughly the same for the age-adjusted and unadjusted thresholds, the coefficient of age was larger when age-related changes were removed from the threshold data. As detailed in Section “Discussion,” this is likely due to age effects, both vestibular and non-vestibular, being removed from the threshold measure, causing a stronger effect of age on the chance of failing condition 4. We compared regression models that included various combinations of age-adjusted thresholds and found that the selected model had the lowest BIC value (these models are detailed in Section “Model Comparisons” in Appendix).

**Table 8:** Results of a multiple logistic regression after application of a stepwise algorithm to provide a simplified model.

	Estimate	SE	*t*-Stat	*p*-Value
(Intercept)	−4.05	1.21	−3.34	0.000834
Age	0.0867	0.0231	3.76	0.000171*
Roll tilt 0.2 Hz	1.75	0.618	2.83	0.004723*

*Analyses were done using age-adjusted, log-transformed thresholds*.

*Signifies statistical significance (*p* < 0.05).

We also examined whether a relationship existed between failing condition 4 and vestibular bias. Since a large bias in any direction may indicate the presence of erroneous sensory information, analyses were performed using the absolute value of bias, normalized by the subject’s threshold. No significant contribution was found from bias in any of the five motion axes (*p* > 0.1), and only age was predictive of the chance of failing (*p* = 0.0006).

#### The Relationship between Principal Components and Modified Romberg Foam Test Performance

Since principal components incorporate contributions from all threshold measures, there is a possibility that a latent variable created by projecting all thresholds into a principal component would be a better predictor of the chance of failing condition 4 than using only the roll 0.2 Hz threshold. To evaluate this, we performed a logistic regression using latent variables created using the first and second principal components. For unadjusted, log-transformed thresholds, we found that the first component alone had a statistically significant relationship with the chance of failing condition 4 (*p* = 0.013), while age (*p* = 0.12) and the second component (*p* = 0.77) did not. Interestingly, a model that included only the first principal component (BIC 85.2) provided a similar fit quality to age + roll tilt 0.2 Hz thresholds (BIC 85.4), and slightly better fit quality than roll tilt 0.2 Hz thresholds alone (BIC 88.0) or age + first component (BIC 86.9). These results suggest that the first principal component includes both vestibular and age effects. We performed a similar analysis for age-adjusted, log-transformed thresholds. We found that age (*p* = 0.00033) and the first component (*p* = 0.010) had a statistically significant relationship with the chance of failing condition 4, while the second component (*p* = 0.65) did not. In this case, a model that included age + first component (BIC 86.1) provided a similar fit quality to age + roll tilt 0.2 Hz thresholds (BIC 84.9), and a better fit quality than the first component alone (BIC 99.8).

## Discussion

We report correlations among the five thresholds measured, and between thresholds, age and the chance of failing condition 4 of the Modified Romberg balance test. We found moderate correlations (0.30–0.51) between vestibular thresholds, even while using age-adjusted thresholds. PCA suggest that lower or higher thresholds across all threshold measures are an individual trait which account for roughly 60% of the variation in the population; this can be further portioned into about 20% of variation being explained by aging and about 40% of the variation being explained by the first component that represents common variations across the five vestibular thresholds measured.

When only roll tilt 0.2 Hz thresholds and age were analyzed together, we found that the chance of failing condition 4 depends significantly on both (*p* = 0.006 and *p* = 0.013, respectively). An analysis incorporating more variables found that the chance of failing condition 4 depended significantly only on roll tilt 0.2 Hz thresholds (*p* = 0.046) and not age (*p* = 0.10), sex, nor any of the other four threshold measures, suggesting that some of the age effect might be captured by the fact that vestibular thresholds increase with age. This contrasts with our published univariate analyses which found a significant correlation with all five thresholds, and did not examine their combined contributions.

Illustrating the importance of considering both age and vestibular thresholds, at 60 years of age the average chance of failing condition 4 is 43%, but this ranges from roughly 5% for the lowest roll tilt thresholds in our population to 80% for the highest roll tilt thresholds. As a second illustration, at the median 0.2 Hz roll tilt threshold, the chance of failing condition 4 is 12%, but this ranges from roughly 3% at 18 years to 50% at 80 years.

We emphasize that these were subjects who qualified as healthy normals. Subjects who reported dizziness, imbalance, or other vestibular symptoms on the mandatory healthy questionnaire were excluded. Thus, we are characterizing how normal variability in human balance depends on “subclinical” intersubject differences in vestibular precision. In this way, our study complements previous work showing that vestibular dysfunction negatively impacts clinical balance test performance (3, 4, 6, 17–20).

### New Contributions of This Study Compared with Our Previous Study

While our previous study (1) investigated some of the same topics as this study, several new and significant results are described herein. The previous study briefly stated that statistically significant correlations existed between threshold measures; this study visualizes these relationships and provides correlation coefficients both for unadjusted and age-adjusted thresholds. The PCA results are new.

The previous study, using single-variable logistic regression, reported that without age adjustment, all five threshold measures had a statistically significant correlation with the chance of failing condition 4 (*p* < 0.007). Even after age adjustment, the previous study reported statistically significant correlations for roll tilt 0.2 Hz thresholds (*p* = 0.003) and roll tilt 1 Hz thresholds (*p* = 0.02), a suggestion of possible correlation with yaw rotation thresholds (*p* = 0.09) and z-translation thresholds (*p* = 0.09), and not significant results for y-translation thresholds (*p* = 0.50). By contrast, the new multiple variable analysis found that only roll tilt 0.2 Hz thresholds and age had a statistically significant contribution to the chance of failing condition 4. The difference between the two results arises because the multiple variable analysis adjusts for the correlations between thresholds, while the single-variable regression fits do not. This study also shows the chance of failing condition 4 for different combinations of age and roll tilt 0.2 Hz thresholds, while the previous study provided an odds ratio for the chance of failing as a function of thresholds only. While the previous analyses showed that threshold was a significant contributor to chance of failing condition 4, even after age adjustment, it did not show the relative contribution of age and roll tilt 0.2 Hz thresholds (Figure 6).

### Interpretation of Correlation Coefficients

Even after age adjustment, the correlation coefficients between different threshold measures ranged from 0.30 to 0.51, which are moderate correlations. We now consider three possibilities for how these correlations may arise from: (1) anatomical variations that impact all peripheral organs similarly, (2) shared peripheral organs, and/or (3) shared processing of cues.

The first principal component included relatively similar contributions from all thresholds, consistent with anatomical variation that impacts all organs or a common central cause. However, this does not explain the differences in correlation coefficients between threshold measures for different motion directions.

Correlations may arise from shared peripheral sensory organs. The largest coefficient (0.51) was between roll tilt 0.2 Hz and roll tilt 1 Hz thresholds, which may be explained by the shared superior and posterior SCC cues. Shared otolith cues may explain the next largest correlations, between roll tilt (both 0.2 and 1 Hz) and Z thresholds (0.46, 0.48), between Y and Z thresholds (0.44) and between roll tilt (both 0.2 and 1 Hz) and Y thresholds (0.41, 0.37). Finally, the lowest coefficients were between yaw thresholds and all others, which may be explained by separate yaw cues from the horizontal SCC. In particular, yaw and roll thresholds had the lowest coefficients (0.30, 0.31), which indicates that even within the SCCs, each canal plane has some unique characteristics. This supports the view that peripheral changes are either a less predominant cause of the variations or may be somewhat different for different end organs.

Correlations may arise from shared processing of vestibular cues. There is substantial evidence for spatial orientation internal models that combine otolith and SCC cues (46–53). In addition, there is evidence that there are separate streams of processing for the components of rotation that are about an earth-vertical vs. earth-horizontal axes (54–56). More specifically, yaw rotations about an earth-vertical axis only receive useful motion cues from the SCC, while tilts and translation about an earth-horizontal axis require SCC–otolith integration to disambiguate tilt and translation. This may result in our earth-vertical yaw rotation thresholds having lower correlations with the other threshold measures, all of which required SCC–otolith integration.

We note that multiple comparisons correction strictly applies to the case of a small number of rejections of the null hypothesis among a large group in which the null hypothesis is not rejected. In our correlations between thresholds, the null hypothesis was rejected in 17 of 20 comparisons, so the remaining three may not have occurred by chance.

### Structure Underlying Variation in Vestibular Thresholds

Principal component analysis found that one component accounts for roughly 60% of the variation in the population. After age adjustment, 21% of variation is explained by aging and 41% of the variation is explained by the first component. The projection of the first component hardly changes after age adjustment, suggesting that lower or higher thresholds across all measures are an important individual trait. A possible interpretation of this component is that is represents (or partially represents) physiologic/biologic age (57, 58), which account for variations not explained by chronologic age (i.e., in years). It could also arise from anatomical or physiological covariation across subjects.

The remaining components (two through five) account for 40% of variation, and are more difficult to interpret, especially since they each explain roughly 10% of the total variation. The second component may allow a decoupling of yaw and roll thresholds, consistent with their low correlation. These components also include measurement imprecision by definition. For example, we have used simulations to estimate that when thresholds are determined using 100 trials and a three-down, one-up staircase, the predicted coefficient of variation is 18% (37). This accounts for only random effects, and does not consider “real world” effects like subjects losing concentration, fatigue and other lapses.

### Balance Test Results

#### From Correlation to Expression of Fundamental Causes

Although correlations do not prove causation, a few factors convince us that the regression results are an expression of roll tilt 0.2 Hz thresholds as a fundamental cause of changes in balance performance. We describe these in detail below, but begin with a summary: (1) the converse relationship, that changes in balance cause changes in vestibular function, seems less likely; (2) among the thresholds we measured, roll tilt 0.2 Hz thresholds assay the cues that provide the most physically relevant sensory feedback for postural control; and (3) if covariate(s) of vestibular thresholds were responsible for the correlation between thresholds and chance of failing condition 4, it is unlikely that they would only covary with roll tilt 0.2 Hz thresholds.

First, while evidence from patients with vestibular deficits shows that vestibular function plays a causal role in the control of balance (3, 4, 6, 17–20), no evidence exists (to the best of our knowledge) that changes in balance cause changes in vestibular function. While vestibular sensory feedback has been shown to affect postural control, it is less clear how changes in balance caused by aging would affect vestibular function.

Second, the physiologic ability to remain upright depends on the sensation of gravitational direction, and thus roll tilt cues would be one of the fundamental cues required to control balance (59, 60). We have shown that roll tilt 0.2 Hz thresholds depend on both otolith cues about the direction of gravity as well as SCC cues about angular rotation (40). Thus, the regression results are consistent with this physical prediction. This argument is strengthened by the lack of statistically significant correlations between chance of failing condition 4 and vestibular cues (e.g., yaw rotation) that do not have a direct theoretical rationale for contributing to postural control.

Third, we consider the possibility that vestibular thresholds are covariates of another variable that is the true primary underlying cause of balance dysfunction, since we did not quantify all of the many contributors to balance (e.g., biomechanics, muscle strength, proprioception, and vision—when available, movement strategies, sensory integration, cognitive processing, etc.) (4). For example, both muscle strength and vestibular function decline with age, and thus are likely correlated across subjects. Since muscle strength is correlated with fall risk (61) and balance performance (62), one could imagine that a regression between vestibular function and balance performance could give a positive result that arises because of covariation between vestibular function and muscle strength. However, if this were true, it is unlikely that only a single vestibular threshold out of five would be correlated with covariates, but we find only a single vestibular threshold is correlated with the chance of failing condition 4. Furthermore, it would be an unlikely coincidence that roll tilt 0.2 Hz threshold would be correlated with the chance of failing condition 4 because of covariates, given the physical explanation described earlier.

We conducted further analyses to provide additional support that covariates are unlikely to explain our correlations. We performed a multiple variable logistic regression between age, Y, Z, yaw, and roll tilt 1 Hz thresholds (i.e. all measures except for roll tilt 0.2 Hz thresholds) and found that none of the thresholds had a statistically significant relationship with the chance of failing condition 4 (Y *p* = 0.71; Z *p* = 0.46; yaw *p* = 0.40; roll tilt 1 Hz *p* = 0.16). We compared this to the multiple variable logistic regression that included the same variables as well as roll tilt 0.2 Hz thresholds. The difference between these fits was statistically significant (χ^2^-statistic, *p* = 0.037) showing that adding the roll tilt 0.2 Hz thresholds as a regression variable yielded significant model fit improvements even after all of the other available variables (including their various covariations) had already been included in the fit. A similar analysis in which each of the four other thresholds was dropped from the model did not yield a statistically significant difference from the model that included the five thresholds (χ^2^-statistic, *p* > 0.39).

#### Sagittal vs. Frontal Plane Motion

Balance requires control of orientation relative to gravity in both the sagittal (anterioposterior) and frontal (mediolateral) planes, and roll tilt thresholds only assay the cues that provide feedback about motion in the frontal plane. Future studies could measure pitch thresholds to determine the cues relevant to sagittal control, and to estimate the relative importance of sagittal and frontal mechanisms to the chance of failing condition 4. There is some evidence that the task we used in which the feet were together primarily challenges frontal plane control, since the rates of failing condition 4 were >20 vs. 1.8% in a similar task with a wide stance (63), which provides less frontal plane challenge and presumably similar sagittal plane challenge. Therefore, we hypothesize that, for the task we used, the chance of failing condition 4 would have a stronger correlation with roll tilt thresholds than pitch thresholds.

#### Model Comparison

A number of models performed similarly well at predicting the chance of failing condition 4, with BIC values between 84.9 and 86.9: (i) first principal component of thresholds, (ii) age + roll tilt 0.2 Hz thresholds, (iii) age + first principal component of age-adjusted thresholds, (iv) age + roll tilt 0.2 Hz age-adjusted thresholds, and (v) age + first principal component of thresholds. Since age + roll tilt 0.2 Hz thresholds relies on less data than PCA, and since it does not require the additional step of age adjustment, the simplicity of this model may make it preferable.

We found that age had a larger coefficient when roll tilt 0.2 Hz thresholds were age-adjusted vs. when they were unadjusted. Since vestibular age effects were removed from the threshold measure, it is unsurprising that this causes age itself to have a stronger effect on the chance of failing condition 4. Furthermore, age serves as a proxy for the various contributors to balance which we did not measure (i.e., non-vestibular). Thus, we propose that the effects of age on vestibular thresholds also covaried with these non-vestibular factors, and when the covarying age effects were removed from the roll tilt 0.2 Hz thresholds, the age coefficient was adjusted to capture just the non-vestibular age effects on the chance of failing condition 4.

Related to this, we also note that while age is statistically significant in the model that includes roll tilt 0.2 Hz thresholds and age, in the models that include age, sex and all five thresholds, age is not statistically significant (*p* = 0.10). The most likely explanation for this is that since vestibular thresholds vary with age, the age effect is spread among age and the vestibular thresholds, decreasing the effect size for each variable and making it harder to reach the level of statistical significance. This explanation is supported by the fact that in the same regression using age-adjusted thresholds (Section “Model Comparisons” in Appendix), age is still statistically significant (*p* = 0.00026). An alternate explanation is that adding additional variables to the model increases variability overall and makes it less likely that each variable will reach statistical significance without a corresponding increase in the number of subjects. Another explanation that we cannot refute at this time is that vestibular thresholds might serve as a biomarker for physiologic/biologic age (57, 58).

We also performed a regression that predicted the chance of failing condition 4 based on age (*p* = 0.42), roll tilt 0.2 Hz threshold (*p* = 0.017) and an interaction term (*p* = 0.054). Since the interaction term did not strictly reach the criteria for statistical significance, we did not present it in the Results. We note that this may have occurred because our population was not extremely large, and because there were not many subjects that were either older with low thresholds, or younger with high thresholds. We speculate that in a study with a larger population, each of these terms might be statistically significant.

We found no evidence that vestibular biases were correlated with the chance of failing condition 4. We can only speculate about the reasons. Perceptual biases may be unrelated to motor errors. The brain may be better at compensating for biases in balance vs. in perception. Alternatively, only large biases may have an effect on balance, and our subjects may not have exhibited large enough biases to demonstrate a relationship. Finally, the measured biases may reflect cognitive processes rather than having a sensory origin (64).

### Unique Aspects of Yaw Rotation

We noted a few ways in which yaw rotation threshold responses differ from those of other motions, in addition to those that have been previously reported; we summarize them here. First, while our previous study presented an age regression that fit well across all five threshold measures, when fits were done to individual threshold measures, only yaw rotation did not have a statistically significant (*p* = 0.087) age effect (1); qualitatively, it also seems that the age cutoff may be higher for yaw rotation. Other studies also reported the lack of statistically significant changes in yaw thresholds with age (65–67). Second, yaw rotation thresholds have the weakest correlation with other threshold measures; specifically, the lowest correlation coefficients were between yaw thresholds and the other four thresholds. Third, velocity storage has different properties for yaw rotation vs. other motions, including less dependence on otolith cues for yaw rotation even with the subject supine (68), and a longer time constant for yaw rotations about an earth-vertical axis (69). Finally, the yaw plane plays a unique role during navigation (70). Different and/or more extensive processing for yaw rotation may result in reduced age effects.

## Brief Summary

We investigated correlations between thresholds and multiple variable correlations between thresholds and the chance of failing condition 4 of the Modified Romberg balance test, which increases vestibular reliance by having subjects stand on foam with eyes closed. We found that the chance of failing condition 4 depends significantly on both roll tilt 0.2 Hz thresholds (*p* = 0.006) and age (*p* = 0.013). We also found moderate correlations (0.30–0.51) among the five vestibular thresholds, even after using our published aging regression to remove most aging effects.

## Ethics Statement

The study was approved by the MEEI Human Studies Committee and written informed consent was obtained from all subjects as dictated by the Declaration of Helsinki.

## Author Contributions

MB, TC, and DM designed the study and collected the data. FK, WW, DM, and TC performed statistical analyses and interpretation. FK drafted the manuscript and WW, DM, TC, and MB provided critical feedback on the manuscript.

## Conflict of Interest Statement

The authors declare that the research was conducted in the absence of any commercial or financial relationships that could be construed as a potential conflict of interest.

## Footnotes

^1^According to the 2010 national vital statistics report (21), the following are the leading causes of death in United States: (1) heart disease (598,000), (2) cancer (575,000), (3) chronic respiratory diseases (138,000), (4) stroke (129,000), (5) accidents (121,000), (6) Alzheimer’s (83,000), (7) diabetes (69,000), (8) kidney diseases (50,000), (9) influenza and pneumonia (50,000), and (10) suicide (38,000). All death rates are rounded to nearest thousand.

## Appendix

### A. Model Comparisons

We investigated a number of logistic regression models to find the most appropriate descriptor of our results (Table A1). Models are listed in ascending order of the BIC, with lower values indicating better models. This comparison shows that there are two categories of models that best describe the data, with none of the five models clearly better than the others. The first is age and roll 0.2 Hz, with or without age adjustment. The second is the first PCA component, with or without the age term included, and with or without age adjustment.

**Table A1:** Comparison of logistic regression models.

Model	*p*-Values of each coefficient	BIC	AIC
1 + Age + roll 0.2 Hz (age adjusted)	0.00017 0.0047	84.9	77.1
1 + First PCA component	0.000075	85.2	80
1 + Age + roll 0.2 Hz	0.013 0.0058	85.4	77.7
1 + Age + first PCA component (age adjusted)	0.00032 0.0087	86.1	78.3
1 + Age + first PCA component	0.098 0.013	86.9	79.1
1 + Roll 0.2 Hz	0.000081	88	82.8
1 + Age + yaw + roll 0.2 Hz	0.014 0.36 0.014	89.2	78.8
1 + Age + roll 1 Hz	0.040 0.036	89.3	81.5
1 + Age + roll 1 Hz + roll 0.2 Hz	0.063 0.42 0.039	89.4	79
1 + Age + sex + roll 0.2 Hz	0.016 0.42 0.0055	89.4	79
1 + First PCA component + second PCA component	0.000087 0.54	89.4	81.6
1 + Age + Z + roll 0.2 Hz	0.063 0.52 0.018	89.6	79.3
1 + Age + Y + roll 0.2 Hz	0.012 0.63 0.0072	89.8	79.4
1 + Age + first PCA component + second PCA component (age adjusted)	0.00033 0.010 0.65	90.5	80.1
1 + Age + Z	0.026 0.090	91.1	83.3
1 + Age + first PCA component + second PCA component	0.12 0.013 0.77	91.4	81
1 + Age + yaw	0.00060 0.11	91.4	83.6
1 + Age + Y	0.0010 0.43	93.4	85.7
1 + Age + Y + roll 1 Hz + roll 0.2 Hz	0.055 0.53 0.37 0.033	93.6	80.6
1 + First PCA component (age adjusted)	0.0052	99.8	94.6
1 + Age + yaw + Y + Z + roll 1 Hz + roll 0.2 Hz	0.084 0.41 0.41 0.75 0.52 0.044	102	83.6
1 + First PCA component + second PCA component (age adjusted)	0.0058 0.63	104	96.4
1 + Age + sex + yaw + Y + Z + roll 1 Hz + roll 0.2 Hz (age adjusted)	0.00026 0.54 0.45 0.46 0.67 0.47 0.047	105	84.3
1 + Age + sex + yaw + Y + Z + roll 1 Hz + roll 0.2 Hz	0.10 0.59 0.48 0.49 0.77 0.50 0.046	106	85.3

*Most models included an intercept term (indicated by 1+). Most analyses used log-transformed thresholds, and some used age-adjusted, log-transformed thresholds (indicated by age adjusted). The second column shows the *p* values for each term, excluding the intercept term. The third and fourth terms show the Bayesian information criterion (BIC) and Akaike information criterion (AIC) for each model, with a lower value indicating a better model*.

*PCA, principal component analysis*.
