# Corrigendum: Multivariate Analyses of Balance Test Performance, Vestibular Thresholds, and Age

**DOI:** 10.3389/fneur.2020.556797

**Published:** 2020-11-26

**Authors:** Faisal Karmali, María Carolina Bermúdez Rey, Torin K. Clark, Wei Wang, Daniel M. Merfeld

**Affiliations:** ^1^Jenks Vestibular Physiology Laboratory, Mass Eye and Ear Infirmary, Boston, MA, United States; ^2^Otolaryngology, Harvard Medical School, Harvard University, Boston, MA, United States; ^3^Smead Aerospace Engineering Sciences, University of Colorado, Boulder, CO, United States; ^4^Division of Sleep Medicine, Brigham and Women's Hospital, Boston, MA, United States

**Keywords:** vestibular, balance, perception, thresholds, aging, multivariate

In the original article, there was an error. We realized there was a typographical error in a sentence of the Results section that describes a previous paper, such that the “y-” and “z-” were reversed.

A correction has been made to *Results, The Relationship between Vestibular Thresholds and Condition 4 of the Modified Romberg Foam Test, Paragraph 1*. The corrected paragraph reads:

Our previous study (1) examined the basic relationship between failing condition 4 of the Modified Romberg test and vestibular thresholds. However, those analyses only looked at the relationship between each individual threshold and the chance of failing the test, without performing multivariate analyses. Specifically, we performed single-variable logistic regressions between failures and each age-adjusted threshold. There were statistically significant correlations with roll 0.2 Hz (*p* = 0.003) and roll 1 Hz (*p* = 0.02) thresholds, a suggestion of possible correlation with yaw 1 Hz (*p* = 0.09) and z-translation 1 Hz (*p* = 0.09) thresholds, and a non-significant correlation for y-translation (*p* = 0.50) thresholds. Given that thresholds may be correlated with each other, a weakness of this analysis was that it did not determine if the covariation between thresholds described above could have resulted in an artifact of some thresholds being correlated with the chance of failing condition 4.

The authors apologize for this error and state that it does not change the scientific conclusions of the article in any way. The original article has been updated.
